# Two bifunctional enzymes from the marine protist *Thraustochytrium roseum*: biochemical characterization of wax ester synthase/acyl-CoA:diacylglycerol acyltransferase activity catalyzing wax ester and triacylglycerol synthesis

**DOI:** 10.1186/s13068-017-0869-y

**Published:** 2017-07-15

**Authors:** Nannan Zhang, Zejing Mao, Ling Luo, Xia Wan, Fenghong Huang, Yangmin Gong

**Affiliations:** 1 Key Laboratory of Biology and Genetic Improvement of Oil Crops, Ministry of Agriculture, Oil Crops Research Institute of Chinese Academy of Agricultural Sciences, Wuhan, 430062 China; 2 Hubei Key Laboratory of Lipid Chemistry and Nutrition, Oil Crops Research Institute of Chinese Academy of Agricultural Sciences, Wuhan, 430062 China

**Keywords:** WS/DGAT, Wax ester, *Thraustochytrium roseum*, Triacylglycerol, Substrate specificity, Kinetic parameters

## Abstract

**Background:**

Triacylglycerols (TAGs) and wax esters (WEs) are important neutral lipids which serve as energy reservoir in some plants and microorganisms. In recent years, these biologically produced neutral lipids have been regarded as potential alternative energy sources for biofuel production because of the increased interest on developing renewable and environmentally benign alternatives for fossil fuels. In bacteria, the final step in TAG and WE biosynthetic pathway is catalyzed by wax ester synthase/acyl coenzyme A (acyl-CoA):diacylglycerol acyltransferase (WS/DGAT). This bifunctional WS/DGAT enzyme is also a key enzyme in biotechnological production of liquid WE via engineering of plants and microorganisms. To date, knowledge about this class of biologically and biotechnologically important enzymes is mainly from biochemical characterization of WS/DGATs from Arabidopsis, jojoba and some bacteria that can synthesize both TAGs and WEs intracellularly, whereas little is known about WS/DGATs from eukaryotic microorganisms.

**Results:**

Here, we report the identification and characterization of two bifunctional WS/DGAT enzymes (designated TrWSD4 and TrWSD5) from the marine protist *Thraustochytrium roseum*. Both TrWSD4 and TrWSD5 comprise a WS-like acyl-CoA acyltransferase domain and the recombinant proteins purified from *Escherichia coli* Rosetta (DE3) have substantial WS and lower DGAT activity. They exhibit WS activity towards various-chain-length saturated and polyunsaturated acyl-CoAs and fatty alcohols ranging from C_10_ to C_18_. TrWSD4 displays WS activity with the lowest *K*_m_ value of 0.14 μM and the highest *k*_cat_/*K*_m_ value of 1.46 × 10^5^ M^−1^ s^−1^ for lauroyl-CoA (C_12:0_) in the presence of 100 μM hexadecanol, while TrWSD5 exhibits WS activity with the lowest *K*_m_ value of 0.96 μM and the highest *k*_cat_/*K*_m_ value of 9.83 × 10^4^ M^−1^ s^−1^ for decanoyl-CoA (C_10:0_) under the same reaction condition. Both WS/DGAT enzymes have the highest WS activity at 37 and 47 °C, and WS activity was greatly decreased when temperature exceeds 47 °C. TrWSD4 and TrWSD5 are insensitive to ionic strength and reduced WS activity was observed when salt concentration exceeded 800 mM. The potential of *T. roseum* WS/DGATs to establish novel process for biotechnological production of WEs was demonstrated by heterologous expression in recombinant yeast. Expression of either TrWSD4 or TrWSD5 in *Saccharomyces cerevisiae* quadruple mutant H1246, which is devoid of storage lipids, resulted in the accumulation of WEs, but not any detectable TAGs, indicating a predominant WS activity in yeast.

**Conclusions:**

This study demonstrates both in vitro WS and DGAT activity of two *T. roseum* WS/DGATs, which were characterized as unspecific acyltransferases accepting a broad range of acyl-CoAs and fatty alcohols as substrates for WS activity but displaying substrate preference for medium-chain acyl-CoAs. In vivo characterization shows that these two WS/DGATs predominantly function as wax synthase and presents the feasibility for production of WEs by heterologous hosts.

**Electronic supplementary material:**

The online version of this article (doi:10.1186/s13068-017-0869-y) contains supplementary material, which is available to authorized users.

## Background

Triacylglycerols (TAGs) are the most common neutral lipids found in animal, plant, and eukaryotic microorganisms as lipid-based energy reserves. In bacteria, polyhydroxyalkanoic acids represent the most abundant class of neutral lipids, which were frequently described to accumulate among hydrocarbon-utilizing marine bacteria [1]. In contrast, storage lipids in the form of TAGs and wax esters (WEs) are less frequently found in prokaryotic bacteria. A few examples show that certain gram-positive bacteria of the actinomycetes group, belonging to the genera *Rhodococcus*, *Mycobacteria*, *Nocardia*, and *Streptomyces*, are able to synthesize and accumulate large quantities of TAGs [2–4]. Gram negative bacteria, such as *Acinetobacter*, and some genera of marine hydrocarbonoclastic bacteria such as *Alcanivorax* usually accumulate WEs together with small amounts of TAGs [5–7]. These lipid-producing microorganisms have high levels of neutral lipids usually under growth limiting condition such as nitrogen starvation [8, 9] or under conditions of unbalanced growth in the presence of abundant carbon source such as gluconate and petroleum hydrocarbons [10].

Microbial TAGs and WEs are major storage lipids accumulated within the cell, provide precursors for chemical industries, and can serve as the feedstock for renewable biofuels. In contrast to TAG consisting of a glycerol backbone esterified by three fatty acyl chains, WE is composed of fatty acid esterified to fatty alcohol, which would not cause the generation of glycerol as an undesirable by-product during the refinement of WEs to biodiesel [11]. Because WEs have a higher energy density than TAG-derived lipids, they could represent a superior feedstock for biodiesel production [12]. Currently, WEs have multiple important technical applications in a variety of areas such as medicine, cosmetics, and food industries, and have the potential for cost-effective and sustainable production of lubricants [12, 13]. Depending on the chain lengths and the degree of unsaturation of fatty acids and fatty alcohol components, WEs have different physical and chemical properties such as melting temperature, oxidation stability, and pressure stability [14]. Liquid WEs consisting of medium to long-chain monounsaturated fatty chains exhibit low melting-points and high oxidation stability, which renders it more suited for lubrication. Currently, the jojoba (*Simmondsia chinensis*) oil is the major biological source of WEs for these commercial applications since the global ban of whale hunting. Unlike other oil storing plant seeds that accumulate large quantities of TAGs as energy reserves, the desert shrub jojoba’s seeds accumulate liquid WEs up to 60% of the seed dry weight, and these WEs are composed mainly C_20:1_ fatty acids and C_20:1_/C_22:1_ fatty alcohols, which generates a carbon chain length of C_38_–C_44_ [15]. However, considering the high price and limited supply of jojoba oil due to the low yield and high cost for cultivating jojoba plant, there is still a strong need for an alternative cost-effective solution for sustainable production of WEs [12]. Recent engineering efforts show that other oilseed plants such as *Camelina sativa* have attracted attention as a promising biological platform form cost-effective production of WEs [12–14, 16, 17].

Reconstruction of WE biosynthetic pathway is required for metabolic engineering of heterologous hosts to produce WEs. The pathway for WE biosynthesis has been well studied in the jojoba plant and certain bacteria that accumulate WEs intracellularly as insoluble inclusions. In *Acinetobacter* sp. strain ADP1, WE biosynthesis is catalyzed by three different enzymes: an NADPH-dependent long-chain acyl-CoA reductase catalyzing the reduction of long-chain acyl-CoA to form the fatty aldehyde intermediate, an unidentified NADPH-dependent fatty aldehyde reductase catalyzing the subsequent reduction of fatty aldehyde to fatty alcohol, and the wax ester synthase/acyl-CoA diacylglycerol acyltransferase (WS/DGAT) that catalyzes the esterification of fatty alcohol and acyl-CoA to form the WE [5, 18]. In contrast to bacteria, a bifunctional NADPH-dependent fatty acyl-CoA reductase directly synthesizes fatty alcohol from acyl-CoA in the jojoba plant, which do not lead to the formation of fatty aldehyde intermediate [6, 19]. In the bacterium *Acinetobacter* sp. strain ADP1, the final step of TAG bioassembly, which involves sequential acylation of the glycerol backbone by three *sn*-specific acyltransferases, is also catalyzed by the WS/DGAT enzyme [5]. The activity of WS/DGAT acting as a bifunctional enzyme is thus shared by both WE and TAG biosynthesis pathways in some bacteria accumulating intracellularly these two different types of neutral lipids at the same time. However, in most microorganisms storing TAGs as the only neutral lipids, the terminal reaction of TAG biosynthesis is usually catalyzed by acyl-CoA:diacylglycerol acyltransferase (DGAT; EC 3.2.1.20) [20]. DGAT transfers an acyl group from acyl-CoA to the *sn*-3 position of 1,2-DAG to form TAG. DGATs form a large family of acyltransferases, and two major types of membrane-bound DGAT enzymes, designated DGAT1 and DGAT2, have been identified in most eukaryotic organisms [21].

Although both DGAT and WS/DGAT possess acyltransferase activity, DGAT enzyme family members do not show protein sequence similarities with the WS/DGAT family members. Moreover, the WS/DGAT enzyme family members share very low overall similarity of amino acid sequences between not only homologs from different species but also homologs within the same species [22]. A number of WS/DGAT enzymes have been identified in plants, animal, and bacteria, with bacterial WS/DGATs representing the largest number of this acyltransferase family [20, 23]. Biochemical characterization of WS/DGATs from bacteria and the jojoba plant shows that WS/DGAT is an rather unspecific enzyme, which is demonstrated by its activity in the WS and/or DGAT reaction towards a broad range of different substrates including saturated and unsaturated acyl-CoAs and fatty alcohols ranging from C_12_ to C_24_ in carbon chain length [24, 25]. This promiscuous characteristic of substrate selectivity of WS/DGATs might be not desirable for specific biotechnological application such as the engineering of WE synthesis in oil crops or microorganisms to produce WEs with tailored chemical composition. However, recent study demonstrates that modification of a specific residue in WS/DGAT enzymes from the bacteria *Marinobacter aquaeolei* VT8 and *Acinetobacter baylyi* could alter fatty alcohol selectivity and kinetic parameters [22]. It shows the potential of rational modification of the already known WS/DGAT enzymes in improving the characteristics of WS/DGAT enzyme activity directed towards specific substrates. On the other hand, the potential for the isolation and discovery of novel genes encoding WS/DGAT enzymes has been recently explored from terrestrial and marine environments [26]. Using molecular method, a number of novel genes encoding putative WS/DGAT proteins from lipid-accumulating bacteria have been identified in marine sediments and soil, which further broadens the diversity of bacterial WS/DGAT enzymes. In contrast to the diversity of WS/DGATs found in a number of bacterial species and the extensive studies on WS/DGATs from bacteria and plants, WS/DGAT enzyme is very rarely reported so far in eukaryotic microorganisms. Here, we report the identification of two bifunctional WS/DGAT proteins from the marine protist *Thraustochytrium roseum*, which may be the first description of WS/DGAT proteins found in marine eukaryotic protist. We also report the biochemical characteristics of these two bifunctional WS/DGAT enzymes with both WS and DGAT activity using in vivo and in vitro assays.

## Results

### Sequence analysis of two putative WS/DGAT enzymes from the protist *T. roseum*

**Fig. 1:**
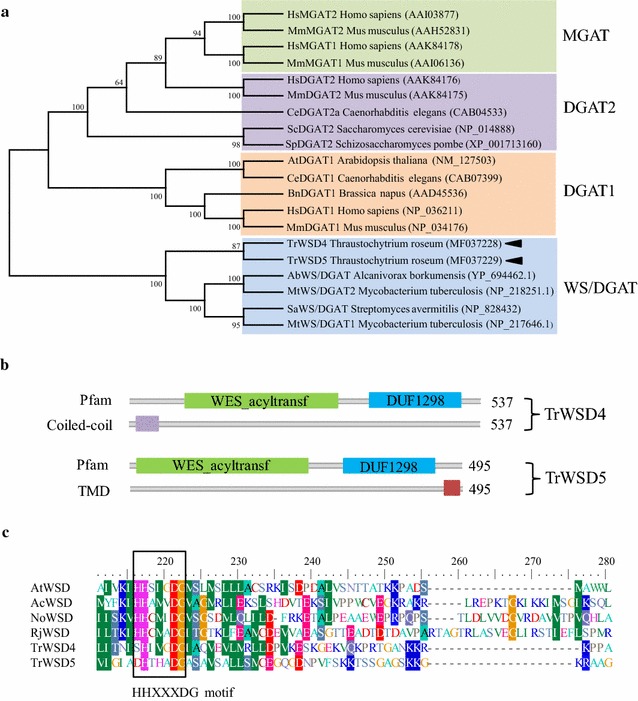
Sequence analysis of *Thraustochytrium roseum* WS/DGAT proteins TrWSD4 and TrWSD5. **a** Phylogenetic relationships among the deduced amino acid sequences of TrWSD4 and TrWSD5 proteins and the MGAT, DGAT1, DGAT2, and WS/DGAT family proteins (*shaded* in *different colors*) from a variety of organisms. The phylogenetic tree was constructed according to the Neighbor-Joining algorithm. GenBank accession numbers are shown by following the corresponding species name. *Numbers at branch points* are bootstrap percentages derived from 1000 replicates. **b** Predicted domains and motifs of TrWSD4 and TrWSD5. The WES_acyltransf (wax ester synthase-like Acyl-CoA acyltransferase) and DUF1298 (protein of unknown function) domains are shown by *green* and *blue squares*. The putative transmembrane domain of TrWSD5 and coiled coil domain of TrWSD4 are shown by *red* and *purple squares*. **c** The conserved HHXXXDG motif of WS/DGAT family members and the substitutions of serine (TrWSD4) and aspartate (TrWSD5) at the first residue of the motif are shown. Domain organization was predicted using HMMER (http://www.ebi.ac.uk/Tools/hmmer/) and the sequence alignment was created with the BioEdit software

*Thraustochytrium roseum* was phylogenetically classified into Thraustochytrids that are a group of marine heterotrophic protists characterized by the presence of an ectoplasmic net, a sagenogenetosome and a cell wall that is composed of non-cellulosic scales [27]. In the fatty acid profile of *T. roseum*, docosahexasaenoic acid (22:6, 46%), palmitic acid (16:0, 26%), oleic acid (18:1, 12%), and stearic acid (18:0, 8%) are the major fatty acids [28]. Having high content of DHA, *T. roseum* was thus considered to be a promising alternative to marine fish oils rich in omega-3 very long-chain polyunsaturated fatty acids. *T. roseum* also accumulates large amounts of triacylglycerols (TAGs) within the cell during the stationary growth phase. We have sequenced the genome of *T. roseum* (ATCC28210), and genome annotation indicates that the *T. roseum* genome encodes at least three putative acyl-CoA:diacylglycerol acyltransferases (DGATs), which catalyze the final step of acyl-CoA-dependent TAG biosynthesis. Full-length cDNA sequences of these three acyltransferases were amplified with RT-PCR based on the corresponding DNA sequences which were retrieved from the assembled *T. roseum* genome sequence. Of the three putative DGATs from *T. roseum*, one shares 100% identity in the deduced amino acid sequence with the previously reported TaDGAT2, which has been identified as a type 2 DGAT enzyme in the oleaginous marine protist *Thraustochytrium aureum* [29]. We performed BLAST against the appropriate GenBank divisions using the deduced amino acid sequences of the other two putative DGAT proteins, and observed a hit of the wax synthase-like acyltransferase domain. These two putative DGAT proteins from *T. roseum* were thus designated TrWSD4 and TrWSD5, and the corresponding coding sequences were deposited in GenBank (TrWSD4 Accession Number: MF037228; TrWSD5 Accession Number: MF037229). Furthermore, we constructed a phylogenetic tree using the amino acid sequences of TrWSD4, TrWSD5 and other 18 acyltransferases representing the different families of DGAT1, DGAT2, WS/DGAT, and MGAT (monoacylglycerol acyltransferase). As shown in Fig. 1a, the 20 acyltransferases from various organisms are clearly divided and grouped into four families, of which both TrWSD4 and TrWSD5 fall into the WS/DGAT family. We postulated that TrWSD4 and TrWSD5 might encode a WS/DGAT enzyme. Further phylogenetic analysis of WS/DGATs from plant, bacteria, and *T. roseum* WS/DGATs showed that *T. roseum* WS/DGATs are separated from other WS/DGATs and form a new cluster consisting of only TrWSD4 and TrWSD5 with 87% bootstrap support (Additional file 1: Figure S1). This observation may suggest a distinct evolutionary origin of *T. roseum* WS/DGATs from that of plant and bacterial WS/DGATs.

Search of protein database using the deduced amino acid sequences of TrWSD4 and TrWSD5 revealed a limited similarity with other WS/DGATs from plant and bacteria (23–29% identity). However, the deduced amino acid sequences of both TrWSD4 and TrWSD5 contain a wax synthase-like acyltransferase domain and a DUF1298 domain with unknown function (Fig. 1b). In addition, the presence of a coiled coil domain at the N-terminus of TrWSD4 was observed and TrWSD5 has a predicted transmembrane region at the C-terminus (residues 472–494). Both TrWSD4 and TrWSD5 contain the conserved acyltransferase active-site motif (HHXXXDG), whose first histidine residue is substituted to serine and aspartate, respectively (Fig. 1c). However, the second histidine residue of the acyltransferase motif is strictly conserved, which is essential for catalytic activity in non-ribosomal peptide bond formation [5].

### Acyl-CoA specificity of TrWSD4 WS activity

**Fig. 2:**
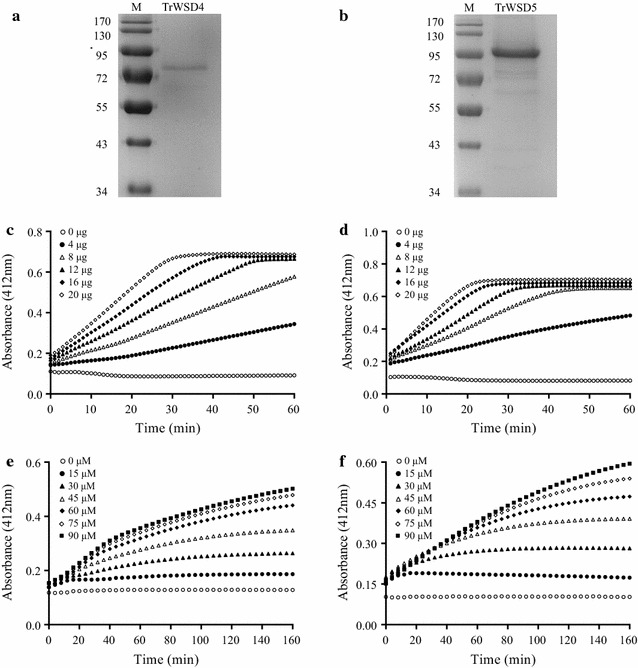
WS activity of recombinant TrWSD4 and TrWSD5 proteins purified from *E. coli*. The purified TrWSD4-6 × His (**a**) and TrWSD5-6 × His (**b**) were subjected to electrophoresis on a 12% Tris–HCl, SDS–polyacrylamide gel. The time-dependent CoA release catalyzed by TrWSD4 (**c**) and TrWSD5 (**d**) was monitored at *A*_412_ for various protein concentrations: 0, 4, 8, 12, 16, and 20 μg. The concentrations of substrates palmitoyl-CoA (90 μM) and hexadecanol (100 μM) were constant. The time-dependent CoA release by 5 μg TrWSD4 (**e**) or 5 μg TrWSD5 (**f**) of varied palmitoyl-CoA concentrations: 0, 15, 30, 45, 60, 75, and 90 μM. The concentration of hexadecanol (100 μM) was constant. Reactions were performed at 37 °C in mixtures including 25 mM sodium phosphate buffer (pH 7.4) and 1 mg/mL DTNB

To examine the biochemical activity of TrWSD4 and TrWSD5 proteins, we overexpressed the *T. roseum* WS/DGAT protein in-frame with a C-terminal 6-histidine (His_6_) tag in *E. coli* Rosetta (DE3) cells. The recombinant proteins of soluble TrWSD4-His_6_ and TrWSD5-His_6_ were purified by Ni^2+^-affinity chromatography. Growing the induced bacterial cultures of 200 mL at 37 °C could allow us to obtain about 200–600 μg of nearly pure soluble proteins (Fig. 2a, b). We first used the purified TrWSD4-His_6_ and TrWSD5-His_6_ to confirm their acyltransferase activity by monitoring the time-dependent increases in *A*_412_. When acyl-CoA and fatty alcohol were used as substrates, WS activity could be measured using the coupled reaction of DTNB and free CoA released during the reaction of esterification of these two substrates [30]. Figure 2c and d shows the influence of various TrWSD4 and TrWSD5 concentrations at a constant concentration of palmitoyl-CoA (90 μM) and hexadecanol (100 μM). Although nearly linear time-dependent increase in *A*_412_ values was observed, absorbance values leveled off during the 60-min time course for TrWSD4 amounts exceeding 12 μg and TrWSD5 amounts exceeding 8 μg, respectively. Although the varied concentrations of TrWSD4 and TWSD5 concentrations were proportional to the rate of increase in *A*_412_ values, the protein concentrations had no influence on maximum *A*_412_ values, which reflected the constant total concentration of palmitoyl-CoA (90 μM) hydrolyzed in the esterification reaction. Figure 2e, f demonstrates the WS activity of *T. roseum* WS/DGATs at fixed amounts of 5 μg as functions of various palmitoyl-CoA concentrations. In contrast to the case of varied protein concentrations in Fig. 2c, d, maximal *A*_412_ values varied in proportion to the concentrations of palmitoyl-CoAs.

We next explored the capability of TrWSD4 to synthesize WEs at 37 °C by catalyzing the esterification of various acyl-CoA molecular species with hexadecanol (16:0-OH) (Additional file 2: Figure S2). Saturation curves for various saturated acyl-CoAs with carbon chain lengths that varied from 8 to 18 are shown in Additional file 2: Figure S2a, b and c. Values of *K*_m_ and *V*_max_, *k*_cat_ and *k*_cat_/*K*_m_ are summarized in Table 1. Unlike the saturation curves of shorter and longer chain acyl-CoAs (i.e., 8:0-CoA, 10:0-CoA, 12:0-CoA, 16:0-CoA, and 18:0-CoA), values of *V*_0_ for myristoyl-CoA increased up to concentration of 15 μM and then declined (Additional file 2: Figure S2b). While the value of *V*_0_ for oleoyl-CoA and palmitoyl-CoA could increase up to the value exceeding 26 nmol/mg/min, the values of *V*_0_ for other acyl-CoAs increased to no more than 15 nmol/mg/min with the increase of substrate concentration (Additional file 2: Figure S2c). Saturation curves for three polyunsaturated fatty acyl-CoAs are shown in Additional file 2: Figure S2d. In contrast to the values of *V*_0_ for those saturated acyl-CoAs, the values of *V*_0_ for 18:3-CoA, 20:4-CoA, and 20:5-CoA were increased in proportion to substrate concentrations. On the basis of this observation, the values of *K*_m_ and *V*_max_ were determined using the modified form of the Michaelis–Menten equation, which incorporates substrate inhibition and are listed together with the values of *k*_cat_ and *k*_cat_/*K*_m_ in Table 1. While the *K*_m_ values for polyunsaturated acyl-CoAs were greatly higher than saturated medium- and long-chain acyl-CoAs, the lowest *K*_m_ value was observed for lauroyl-CoA (C_12:0_), which suggests a strong preference of TrWSD4 for lauroyl-CoA.

**Table 1:** Kinetic parameters for TrWSD4-catalyzed esterification of hexadecanol and different acyl-CoAs

Substrate	*K*_m_ (μM)	*V*_max_ (nmol/min per mg)	*k*_cat_ (s^−1^)	*k*_cat_/*K*_m_ (M^−1^ s^−1^)
Octanoyl-CoA (C_8:0_)	29.93	14.76	1.85 × 10^−2^	6.19 × 10^2^
Decanoyl-CoA (C_10:0_)	17.6	12.24	1.54 × 10^−2^	8.73 × 10^2^
Lauroyl-CoA (C_12:0_)	0.14	16.08	2.02 × 10^−2^	1.46 × 10^5^
Myristoyl-CoA (C_14:0_)	3.94	20.22	2.54 × 10^−2^	6.44 × 10^3^
Palmitoyl-CoA (C_16:0_)	3.08	25.49	3.20 × 10^−2^	1.04 × 10^4^
Stearoyl-CoA (C_18:0_)	19.15	30.23	3.79 × 10^−2^	1.98 × 10^3^
Octadecatrienoyl-CoA (C_18:3_)	280.86	18.80	2.36 × 10^−2^	8.40 × 10^1^
Arachidonoyl-CoA (C_20:4_)	297.17	11.82	1.48 × 10^−2^	4.99 × 10^1^
Eicosapentaenoyl-CoA (C_20:5_)	131.16	9.05	1.14 × 10^−2^	8.66 × 10^1^

### Acyl-CoA specificity of TrWSD5 WS activity

We also tested the capability of TrWSD5 to synthesize WE at 37 °C by catalyzing the esterification of various acyl-CoA molecular species with hexadecanol (Additional file 3: Figure S3). Saturation curves for various saturated acyl-CoAs with chain lengths that varied from 8 to 18 are shown in Additional file 3: Figure S3a–c. Values of *K*_m_ and *V*_max_, *k*_cat_ and *k*_cat_/*K*_m_ are summarized in Table 2. By contrast to the saturation curves for other acyl-CoAs, an effect of substrate inhibition was observed for oleoyl-CoA. The value of *V*_0_ for oleoyl-CoA increased up to concentration of 30 μM and then declined (Additional file 3: Figure S3c). The increase in the values of *V*_0_ for short-chain acyl-CoAs (C_8:0_ and C_10:0_) only occurred with the increase in the substrate concentration until it reaches saturated at 45 μM (Additional file 3: Figure S3a). The maximum of the value of *V*_0_ was observed up to 179 nmol/mg/min for myristoyl-CoA (C_14:0_) and 165 nmol/mg/min for palmitoyl-CoA (C_16:0_) when substrate concentration reaches 90 and 75 μM, respectively (Additional file 3: Figure S3b, c). Saturation curves for three polyunsaturated fatty acyl-CoAs are shown in Additional file 3: Figure S3d and e. The values of *V*_0_ for 18:3-CoA, 20:4-CoA, and 20:5-CoA were linearly increased in proportion to the increase in substrate concentration. Based on *V*_*i*_ of the WS activity towards various saturated and polyunsaturated acyl-CoAs at different substrate concentrations, kinetic parameters for WS activity of TrWSD5 were calculated and listed in Table 2. Similar to that of TrWSD4, the highest *K*_m_ values were observed for the three polyunsaturated acyl-CoAs. The lowest *K*_m_ value was observed for decanoly-CoA (C_10:0_), indicating the preference of TrWSD5 for this medium-chain acyl-CoA species.

**Table 2:** Kinetic parameters for TrWSD5-catalyzed esterification of hexadecanol and different acyl-CoAs

Substrate	*K*_m_ (μM)	*V*_max_ (nmol/min per mg)	*k*_cat_ (s^−1^)	*k*_cat_/*K*_m_ (M^−1^ s^−1^)
Octanoyl-CoA (C_8:0_)	12.25	63.92	1.02 × 10^−1^	8.33 × 10^3^
Decanoyl-CoA (C_10:0_)	0.96	58.91	9.41 × 10^−2^	9.83 × 10^4^
Lauroyl-CoA (C_12:0_)	25.93	151.9	2.43 × 10^−1^	9.37 × 10^3^
Myristoyl-CoA (C_14:0_)	18.5	207.4	3.31 × 10^−1^	1.79 × 10^4^
Palmitoyl-CoA (C_16:0_)	24.77	213.4	3.41 × 10^−1^	1.37 × 10^4^
Stearoyl-CoA (C_18:0_)	75.87	378.5	6.04 × 10^−1^	7.96 × 10^3^
Octadecatrienoyl-CoA (C_18:3_)	284.68	151.52	2.42 × 10^−1^	8.50 × 10^2^
Arachidonoyl-CoA (C_20:4_)	226.35	103.09	1.65 × 10^−1^	7.29 × 10^2^
Eicosapentaenoyl-CoA (C_20:5_)	106.2	311.7	4.98 × 10^−1^	4.69 × 10^3^

### Effect of temperature on WS activity of TrWSD4 and TrWSD5

**Fig. 3:**
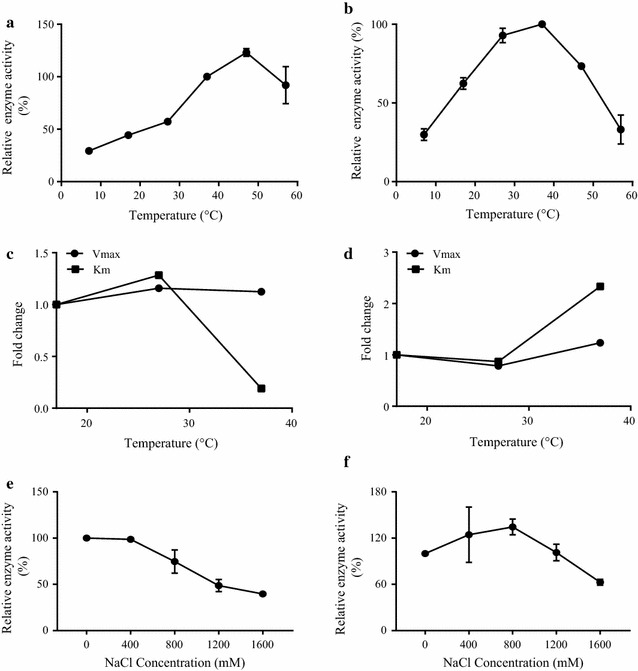
Effects of temperature and ionic strength on WS activity of *Thraustochytrium roseum* WS/DGAT proteins TrWSD4 and TrWSD5. The effect of temperature on the relative WS activity of TrWSD4 (**a**) and TrWSD5 (**b**) was observed. WS activity was determined with the substrates of 90 μM palmitoyl-CoA and 100 μM hexadecanol. The relative WS activity was calculated at different temperatures by taking the WS activity at 37 °C as 100%. The influence of temperature on the values of kinetic parameters *K*_m_ and *V*_max_ for TrWSD4- (**c**) and TrWSD5- (**d**) catalyzed reaction was observed. The value of fold change was calculated at different temperatures by taking the value at 17 °C as 1.0. Reactions were performed in mixtures including 25 mM sodium phosphate buffer (pH 7.4) and 1 mg/mL DTNB. The effect of ionic strength on the WS activity of TrWSD4 (**e**) and TrWSD5 (**f**) was observed at the reaction temperature of 37 °C. WS activity was determined with the substrates of 90 μM decanoyl-CoA and 100 μM hexadecanol for a wide range of NaCl concentrations relative to the reaction without addition of NaCl. Reactions were performed at 37 °C in mixtures including 25 mM sodium phosphate buffer (pH 7.4), 1 mg/mL DTNB and different concentrations of NaCl. WS activity values are averages of a representative experiment performed in triplicate and error bars correspond to one standard deviation (S.D.)

The effect of different temperatures was observed on the WS enzyme activity of both TrWSD4 and TrWSD5. The *V*_i_ was calculated at all six different temperatures (7, 17, 27, 37, 47, and 57 °C) and plotted against the temperatures. The graph shows a regular pattern of enzymatic reaction, which is optimal at the temperature of 47 and 37 °C for TrWSD4 and TrWSD5 (Fig. 3a, b), respectively. The WS activity of TrWSD4 was gradually increased when the temperature was increased from 7 to 47 °C, while decreased to 92% of the highest enzyme activity when the temperature reaches 57 °C (Fig. 3a). By contrast, the lowest WS activity of TrWSD5 was observed at the lowest and the highest temperatures tested in this assay. The enzyme activity of TrWSD5 appears to be stable when the temperature increased from 17 to 47 °C (Fig. 3b). These data suggest that higher temperature or lower temperature inhibits the WS enzyme activity of both TrWSD4 and TrWSD5, and the optimal temperature for a high WS activity of these two WS/DGAT enzymes may be 37 °C. We further characterized the kinetics of WS activity of *T. roseum* WS/DGATs at temperatures ranging from 17 to 37 °C (Fig. 3c, d; Additional file 4: Figure S4a, b). Additional file 4: Figure S4a, b demonstrates the influence of increasing temperature on saturation curves using palmitoyl-CoA and hexadecanol as substrates. Figure 3c, d shows the influence of temperature on the *K*_m_ and *V*_max_. The values of *V*_max_ were not greatly influenced by increasing temperature, while the temperature had substantial influence on the *K*_m_ values. The value of *K*_m_ for TrWSD4 decreased five-fold, whereas the value of *K*_m_ for TrWSD5 increased 2.3-fold when the temperatures increased from 17 to 37 °C.

### Effect of ionic strength on WS activity of TrWSD4 and TrWSD5

To further characterize the WS activity of TrWSD4 and TrWSD5, we tested the effect of increasing salt concentration on the WS enzyme activity. The influence of NaCl concentration on the WS activity was studied using different NaCl concentrations (0, 400, 800, 1200, 1600 mM) in the assay buffer. The WS activity at different NaCl concentrations was measured and relative enzyme activity was calculated. The WS activity of TrWSD4 was gradually declined with the increase in NaCl concentration from 0 to 1.6 M, and the WS activity was almost influenced when the NaCl concentration was increased up to 400 mM (Fig. 3e). The increase in the WS activity of TrWSD5 was observed from 0 to 800 mM NaCl concentration while afterwards from 800 to 1600 mM a gradual decrease in the WS activity was observed (Fig. 3f). TrWSD4 and TrWSD5 show the maximum WS enzyme activity at NaCl concentrations of 400 and 800 mM, respectively. The results indicate the ionic strength-dependent variation in the WS activity of TrWSD4 and TrWSD5 and the relative stability of the WS activity when salt concentration is not in excess of 400 mM for TrWSD4 and 800 mM for TrWSD5.

### Alcohol specificities of *T. roseum* WS/DGATs

**Fig. 4:**
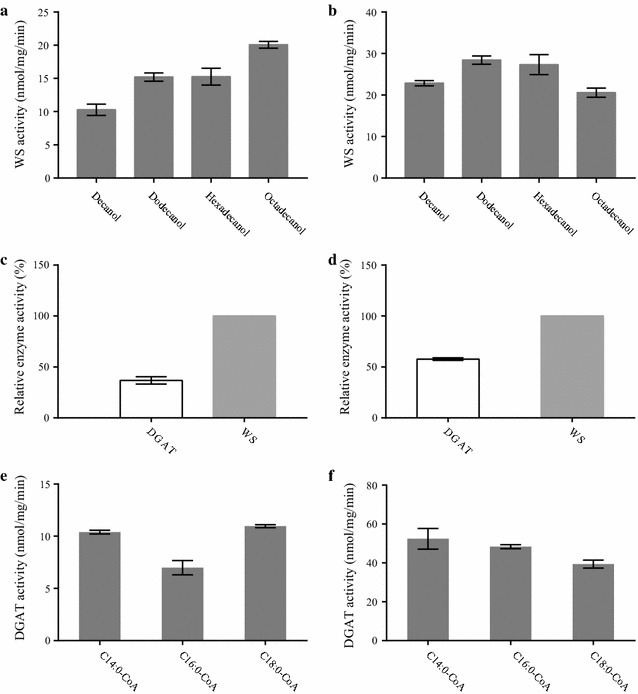
Substrate specificity of WS and DGAT activities of *Thraustochytrium roseum* WS/DGAT proteins TrWSD4 and TrWSD5. Alcohol specificity of WS activity of TrWSD4 (**a**) and TrWSD5 (**b**) was determined by using the substrates of 30 μM palmitoyl-CoA and 100 μM linear alcohols of different chain lengths. Reactions were performed at 37 °C in mixtures including 25 mM sodium phosphate buffer (pH 7.4) and 1 mg/mL DTNB. The DGAT activity of TrWSD4 (**c**) and TrWSD5 (**d**) relative to WS activity of the corresponding recombinant enzyme is shown as percentage. The purified recombinant enzymes were used for WS and DGAT activity assays with the substrates of 15 μM palmitoyl-CoA together with 100 μM hexadecanol (WS activity) or 100 μM DAG (DGAT activity). Acyl-CoA specificity of DGAT activity of TrWSD4 (**e**) and TrWSD5 (**f**) was determined by using the substrates of DAG (100 μM) and acyl-CoAs with different chain lengths. Reactions were performed at 37 °C in mixtures including 25 mM sodium phosphate buffer (pH 7.4) and 1 mg/mL DTNB. Data represent mean values of three replicates ±S.D

We also detected WS activity of both *T. roseum* WS/DGATs using palmitoyl-CoA and different linear alcohols as substrates. TrWSD4 accepted four linear alcohols as substrates and the highest WS activity was observed for octadecanol (C_18:0_-OH), which is two-fold higher than the activity using decanol as substrate (C_18:0_-OH) (Fig. 4a). Four linear alcohols were also utilized by TrWSD5 with almost equal activity (Fig. 4b). These data demonstrate that *T. roseum* WS/DGATs have WS activity towards a broad range of medium- and long-chain linear alcohols.

### DGAT activity of *T. roseum* WS/DGATs

To verify that TrWSD4 or TrWSD5 is indeed a bifunctional enzyme with WS and DGAT activity, we first used the recombinant proteins purified from *E. coli* to measure their DGAT activity. We tested the DGAT activity by monitoring the change of *A*_412_ when the purified recombinant protein was added in the assay in the presence of palmitoyl-CoA and DAG (16:0/16:0), which is different from the measurement of the WS activity when palmitoyl-CoA and 16:0-OH were used as substrates. As shown in Fig. 4c and d, the DGAT activity of TrWSD4 was 33% of the WS activity (Fig. 4c), while the DGAT activity of TrWSD5 was 58% of its WS activity (Fig. 4d). This result demonstrates that both TrWSD4 and TrWSD5 exhibit a WS activity as well as a lower DGAT activity. We also measured the DGAT activity of both *T. roseum* WS/DGATs using DAG and different acyl-CoAs as substrates. As shown in Fig. 4e, f, both TrWSD4 and TrWSD5 exhibited comparable DGAT activity towards medium- and long-chain saturated acyl-CoAs.

### In vivo characterization of TrWSD4 and TrWSD5 using heterologous expression in yeast

**Fig. 5:**
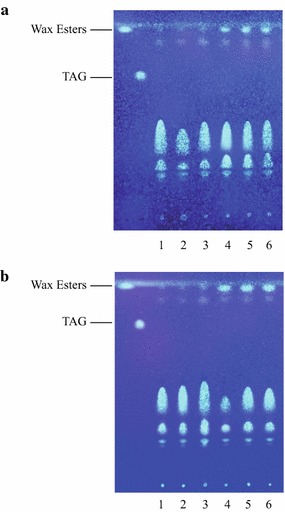
TLC analysis of neutral lipids synthesized in recombinant yeast mutant H1246. **a** WE synthesis in recombinant yeast H1246 expressing TrWSD4. WEs and TAG are shown as reference lipid standards. *1*–*3*, lipid samples extracted from three independent yeast transformants H1246:pESC-URA fed with 0.1% (w/v) palmitic acid together with 0.1% (w/v) hexadecanol; *4*–*6*, lipid samples extracted from three independent yeast transformants H1246:TrWSD4 fed with 0.1% (w/v) palmitic acid together with 0.1% (w/v) hexadecanol. **b** WE synthesis in recombinant yeast H1246 expressing TrWSD5. WEs and TAG are shown as reference lipid standards. *1*–*3*, lipid samples extracted from three independent yeast transformants H1246:pESC-URA fed with 0.1% (w/v) palmitic acid together with 0.1% (w/v) hexadecanol; *4*–*6*, lipid samples extracted from three independent yeast transformants H1246:TrWSD5 fed with 0.1% (w/v) palmitic acid together with 0.1% (w/v) hexadecanol

We further tested the ability of TrWSD4 and TrWSD5 to synthesize WEs or TAG by heterologous expression in the yeast (*Saccharomyces cerevisiae*) mutant H1246 which is deficient in storage lipid biosynthesis [31]. We cloned the TrWSD4 or TrWSD5 into the yeast expression vector pESC-Ura and induced its expression by the addition of galactose in the presence of palmitic acid and hexadecanol in the yeast culture. TLC analysis of the lipids extracted from the recombinant yeast harboring the empty pESC-URA plasmid revealed that these cells accumulated neither WEs nor triacylglycerols (TAGs) when supplemented with palmitic acid together with hexadecanol (Fig. 5a, lanes 1–3). In contrast, the formation of WEs was observed when yeast cells were cultured in inducible medium containing palmitic acid together with hexadecanol (Fig. 5a, lanes 4–6). Similar results were observed for the yeast cells expressing TrWSD5 (Fig. 5b). Unlike WE biosynthesis, heterologous expression of either TrWSD4 or TrWSD5 did not lead to the formation of detectable amounts of TAGs under any conditions tested in our study. We isolated and purified WEs generated in these experiments from yeast total lipids by preparative TLC and determined their molecular structures using GC–MS analysis. Heterologous expression of TrWSD4 led to the formation of WEs consisting of hexadecyl myristate (C_30_) and hexadecyl palmitate (C_32_) (Additional file 5: Figure S5a, b), whereas WEs isolated from total lipids of recombinant yeast expressing TrWSD5 contained hexadecyl laurate (C_28_), hexadecyl myristate (C_30_) and hexadecyl palmitate (C_32_) as well as ethyl palmitate (Additional file 5: Figure S5c–f). The presence of C_28_ and C_30_ WEs as wells as ethyl palmitate in addition to hexadecyl palmitate in the WE products indicates that *T. roseum* WS/DGATs not only utilized exogenously supplemented palmitic acid as substrate but also had the capability to utilize lauryol-CoA (C_12:0_), myristoyl-CoA (C_14:0_), and even ethanol, which were from lipid and glucose metabolism in host yeast, leading to the formation of a WE mixture.

## Discussion

In this study, we identified two WS/DGAT proteins from the marine protist *Thraustochytrium roseum* which possess both acyl-CoA-dependent WS and DGAT activities. The protist *Euglena gracilis* was found to accumulate WEs under anaerobic conditions [32] but the putative WS/DGAT enzyme has not been reported so far. Our study may thus represent the firstly identified bifunctional WS/DGAT acyltransferases from eukaryotic microorganisms. Despite the low similarity in amino acid sequences of these two WS/DGAT proteins with other reported WS/DGAT proteins, the highly conserved acyltransferase domain HHXXXDG is found in the N-terminal regions of TrWSD4 and TrWSD5 (Fig. 1c). Substitution of the first conserved histidine in the acyltransferase domain with serine in TrWSD4 and aspartate in TrWSD5 may not affect their WS and DGAT acyltransferase activities as our in vitro activity assay using their purified combinant enzymes shows their bifunctional acyltransferase activities (Figs. 2, 4). Similar substitution was also observed in other WS/DGATs from bacteria such as *Mycobacteria tuberculosis* RV2484c, *Mycobacteria smegmatis* Wdh3563-3, and *Streptomycetes* SCO1280 [33]. Mutagenesis of serine in TrWSD4 or aspartate in TrWSD5 to the conserved histidine may show whether the first residue in the conserved domain is responsible for some enzymatic properties such as substrate selectivity of WS/DGAT. Further sequence analysis based on phylogeny of the WS/DGAT family of acyltransferases suggests that these two *Thraustochytrium* WS/DGAT proteins form a new branch of WS/DGAT acyltransferases, which may have a different origin from those bacterial WS/DGATs.

The identity of TrWSD4 and TrWSD5 was further supported by in vitro assay using the purified recombinant proteins expressed in *E. coli* and functional expression study in the yeast mutant H1246. Both in vitro assay and in vivo characterization demonstrated the WS activity of these two *Thraustochytrium* WS/DGAT proteins, whereas only in vitro assay showed their DGAT activity since no any detectable amounts of TAG were observed in recombinant yeast mutant expressing either TrWSD4 or TrWSD5 (Fig. 5). Similarly, the lack of in vivo DGAT activity in yeast was also found for a number of WS/DGAT proteins including Arabidopsis WSD1 [34], PhWS1 from petunia [35], and the majority of the 15 WS/DGAT homologs from *Mycobacterium tuberculosis* H37Rv [33]. These similar results suggest that expression of WS/DGAT in yeast could drive fatty acyl flux towards the production of WE rather than TAG. In contrast to these WS/DGAT proteins showing no in vivo DGAT activity, heterologous expression of the *A. calcoaceticus* WS/DGAT enzyme in the yeast quadruple mutant H1246 restored TAG biosynthesis [36], indicating that not all the members of the WS/DGAT family lack in vivo DGAT activity. Previous report shows that expression of *Thraustochytrium aureum* type 2 DGAT (TaDGAT2), which shares 100% amino acid sequence identity with one DGAT annotated in the *T. roseum* genome, restored TAG biosynthesis in the yeast mutant H1246 and displayed in vivo DGAT activity towards long-chain saturated and unsaturated acyl-CoAs and DAG substrates [29]. The presence of a TaDGAT2 homolog with 100% identity in *T. roseum* indicates that at least one DGAT protein other than TrWSD4 and TrWSD5 may be involved in TAG biosynthesis in *T. roseum* in a physiological context. It is not known whether TrWSD4 and TrWSD5 have in vivo DGAT activity in *T. roseum* and contribute to TAG biosynthesis so far.

Biochemical characterization of *T. roseum* WS/DGATs shows that both TrWSD4 and TrWSD5 display WS activity towards various acyl-CoAs of carbon chain length ranging from 8 to 20, which is consistent with the broad substrate profiles of the previously characterized WS/DGATs from bacteria and plants. For example, the jojoba WS has significant activity towards both saturated and monounsaturated acyl-CoAs ranging from 14 to 24 carbons in length [24]. WS/DGAT from *Acinetobacter* sp. Strain ADP1 also accepts different acyl-CoAs of various chain-lengths ranging from 2 to 20 with the highest activity towards palmitoyl-CoA [37]. Our in vivo characterization of TrWSD5 demonstrated the presence of certain amounts of ethyl palmitate in addition to major WE products in lipids extracted from recombinant yeast H1246 (Additional file 5: Figure S5). This result suggests that substrates utilized by *T. roseum* WS/DGATs are probably not limited to those acyl-CoAs tested in our in vitro assays but have a broader range. However, our in vitro assays demonstrate the difference of substrate preference between *T. roseum* WS/DGATs and the previously identified plant and bacterial WS/DGATs. Based on simple comparisons of kinetic features such as *K*_m_ and *k*_cat_/*K*_m_ (Tables 1, 2), lauroyl-CoA (C_12:0_) is the best substrate for TrWSD4, while decanoyl-CoA (C_10:0_) is the most preferred substrate for TrWSD5. The *k*_cat_/*K*_m_ values of TrWSD4 for lauroyl-CoA are four orders of magnitude higher than that for polyunsaturated acyl-CoAs, and the *k*_cat_/*K*_m_ values of TrWSD5 for decanoyl-CoA are two orders of magnitude higher than that for polyunsaturated acyl-CoAs. These observations suggest that medium and short chain acyl-CoAs may be the most preferred acyl-CoA substrates for WS activity of these two *T. roseum* WS/DGATs, which is in contrast to the preference of long-chain acyl-CoAs by plant and bacterial WS/DGATs. There are few examples that reported kinetic parameters of WS/DGATs for different acyl-CoAs with the exception of WS/DGAT from *Acinetobacter* sp. Strain ADP1. The WS reaction catalyzed by *Acinetobacter* sp. WS/DGAT with hexadecanol and different concentrations of palmitoyl-CoA as substrates was found to follow Michaelis–Menten kinetic [37]. The *K*_m_ value for palmitoyl-CoA of 29 μM is comparable to the *K*_m_ value (24.77 μM) of TrWSD5-catalyzed WS reaction for palmitoyl-CoA, while is ninefold higher than that of TrWSD4-catalyzed WS reaction (3.08 μM). In most literatures, substrate specificity of WS/DGATs towards acyl-CoAs was determined based on the comparison of WS activity for various-chain-length acyl-CoAs. Our results showed that although the values of *V*_max_ for different acyl-CoAs were comparable, significant difference in the values of *K*_m_ and *k*_cat_/*K*_m_ for both TrWSD4- and TrWSD5-catalyzed WS reaction was observed among acyl-CoAs with different chain length and saturation (Tables 1, 2). Although our in vitro experiments may not reflect the true kinetic parameters of TrWSD4 and TrWSD5 in the cellular environment, a direct comparison of these parameters can be made as these in vitro experiments were performed using the purified proteins at the same reaction conditions. Our results should reveal the real difference in substrate specificity of these two WS/DGAT enzymes functioning in vivo. TrWSD5 has a predicted membrane-spanning region at the C-terminus, suggesting that this WS/DGAT enzyme may be associated with the membrane. However, the conserved acyltransferase motif (HHXXXDG) which is important for catalytic activity of WS/DGAT is located at the hydrophilic N-terminus of TrWSD5 (Fig. 1b, c); thus, acyl-CoAs are still likely to be soluble physiological substrate for membrane-associated TrWSD5. The lack of a predicted membrane-spanning region in TrWSD4 indicates that it is not probably a transmembrane protein. When *T. roseum* was grown on the medium with glucose as carbon source, no detectable amounts of WEs were observed in total lipids (data not shown). Moreover, annotation of the *T. roseum* genome reveals the absence of a putative fatty acyl reductase which catalyzes the formation of fatty alcohol in WE biosynthesis. These information might indicate that fatty alcohol is unlikely the physiological substrate for these two WS/DGAT enzymes from *T. roseum*. Based on these analyses, we conclude that there is a difference in protein hydrophobility and enzymatic properties between TrWSD4 and TrWSD5.

We further summarized the biochemical properties of *T. roseum* WS/DGATs (TrWSD4 and TrWSD5) and other WS/DGATs characterized so far from prokaryotes and eukaryotes (Additional file 6: Table S1). The biochemical properties of WS/DGATs have been conventionally studied through in vitro enzyme assays which monitor the release of free CoA or the formation of radiolabeled products (WEs or TAGs). In these assays, either radiolabeled or non-radiolabeled acyl-CoAs are used as substrates in reactions catalyzed by purified enzyme, crude extracts of recombinant *E. coli* or yeast strains, or cellular fraction such as microsomes [22, 25, 30, 34]. As shown in Additional file 6: Table S1, WS/DGATs from different organisms vary in size, sequence and hydrophobility, and they have a very broad substrate range, accepting fatty acyl-CoAs with various degrees of saturation and carbon chain length as substrates [36–41]. Alcohol substrates utilized by WS/DGATs include linear fatty alcohols with various chain lengths, branched alcohols, cyclic and aromatic alcohols, and other alcohols with special structure. WS/DGATs also accept mono- and diacylglycerides as substrates. Moreover, some WS/DGATs show the highest activity towards specific acyl-CoA or alcohol. It should be noted that WS/DGAT may have different substrate specificities depending on the expression host and enzyme source used for measurement of enzyme activity and the specific combination of substrates [25].

In contrast to the absence of fatty acid ethyl ester in WE products extracted from recombinant *S. cerevisiae* expressing TrWSD4, functional expression of TrWSD5 in yeast led to the formation of a mixture of biodiesel (ethyl palmitate) and WEs. Shi et al. evaluated the potential of five WS enzymes from different source organisms to engineer *S. cerevisiae* to produce biodiesel [38], which suggests WS acyltransferase activity on acyl-CoA and ethanol. In our study, *T. roseum* WS/DGATs exhibited the predominant WS activity in recombinant yeast, leading to the accumulation of WEs as major products. *T. roseum* WS/DGATs with the highest preference for medium-chain acyl-CoAs would be used to produce WEs with shorter chain lengths via metabolic engineering. To tailor the composition of WEs in suitable hosts, co-expression of appropriate acyl-ACP thioesterases and FARs in combination with *T. roseum* WS/DGATs is likely required for producing high levels of novel WEs. For example, the selection of a 10:0-ACP thioesterase from *Cuphea hookeriana* to modulate the composition of fatty acids in the acyl-CoA pool is likely to redirect the synthesis of acyl-CoAs which is well matched with the preference of TrWSD5 WS activity towards decanoyl-CoA. Similarly, directing the flux of substrate acyl-CoAs towards TrWSD4 with the preference for lauroyl-CoA will require co-expression of a 12:0-ACP thioesterase from *Umbellularia californica* [16]. It is notable that blocking or minimizing TAG synthesis may also be required for future metabolic engineering efforts to accumulate high levels of WE products within the host cells.

## Conclusions

We identified two bifunctional WS/DGATs from the marine protist *T. roseum*, which may represent the first report on WS/DGAT enzymes from the eukaryotic protist. In vitro assays using the purified recombinant enzymes from *E. coli* and in vivo characterization through functional expression in yeast have been performed to confirm their WS and DGAT activities and determine their substrate specificities. Similar to previously identified plant and bacterial WS/DGATs, *T. roseum* WS/DGATs are also unspecific enzymes accepting a broad range of various-chain-length acyl-CoAs and fatty alcohols as substrates. However, kinetic characterization shows their preference of medium-chain acyl-CoAs for WS activity. The use of *T. roseum* WS/DGATs in combination with different FARs has the potential to engineer suitable hosts such as oilseed crops to achiever a synthesis of novel WEs with tailored composition. Our work presents the biochemical properties of two WS/DGATs from the oleaginous protist and provides the basis for reconstruction of WE biosynthetic pathway in heterologous host, but further much more work is needed in order to investigate the potential of utilizing these *T. roseum* WS/DGATs to produce high levels of novel WEs through metabolic engineering approach.

## Methods

### Strains and growth conditions

The marine protist *T. roseum* ATCC28210 was obtained from the American Type Culture Collection (ATCC, Rockville, MD, USA). The culture of *T. roseum* was maintained on 3% agar slants containing the basal medium supplemented with 2% glucose (w/v) and 0.2% yeast extract (w/v). The basal medium contained (g/L): NaCl, 25; MgSO_4_·7H_2_O, 5; KCl, 1; KH_2_PO_4_, 0.1; CaCO_3_, 0.2; (NH_4_)_2_SO_4_, 0.2; NaHCO_3_, 0.1; monosodium glutamate, 2.0. *T. roseum* cells were grown in the medium at 25 °C with orbital shaking at 180 rpm.

The *Saccharomyces cerevisiae* quadruple mutant H1246 *MATα* (*dga1*Δ *lro*Δ *are1*Δ *are2*Δ), gift from Antoni Banas, University of Gdansk and Medical University of Gdansk (Poland), was grown at 28 °C in YPD medium containing 1% (w/v) yeast extract, 2% (w/v) peptone, and 2% (w/v) glucose. Its derivative strains for heterologous expression of the empty vector pESC-Ura, TrWSD4, or TrWSD5 were grown at 28 °C in synthetic complete medium lacking uracil and containing 0.67% (w/v) yeast nitrogen base with ammonium sulfate and 2% glucose.

*Escherichia coli* DH5α was used for cloning and cultured at 37 °C in Luria–Bertani broth with the 100 μg/mL ampicillin. *E. coli* Rosetta (DE3) was used as the expression host for the overproduction of recombinant TrWSD4 and TrWSD5. All strains used in this study are listed in Additional file 7: Table S2.

### RNA isolation and reverse transcription PCR

Total RNA was isolated from the cultures of *T. roseum* using Trizol reagent (Invitrogen) according to the manufacturer’s instruction. Two micrograms of RNA was reverse transcribed and used for cDNA synthesis using the PrimeScript™ Double Strand cDNA Synthesis Kit (Takara) as described by the manufacture. The PCR products were cloned into pMD19-T Simple vector for DNA sequencing.

### Phylogenetic analysis

All protein sequences used for phylogenetic analysis were obtained from the public database at NCBI (http://www.ncbi.nlm.nih.gov/). The phylogenetic tree was constructed after protein sequence alignments using the neighbor-joining (NJ) method which was performed with MEGA 4.0 [42]. The molecular distance of the aligned sequences was calculated according to the poisson correction model. All gap and missing data in the alignments were accounted for by pairwise deletion. Branch points were tested for significance by bootstrapping with 1000 replications. The organisms and GenBank accession numbers for proteins used for phylogenetic analyses are shown on the corresponding phylogenetic trees.

### Construction of plasmids for enzyme overproduction in *E. coli*

Sequence information from annotation of the *T. roseum* genome was used to design cDNA primers. After obtaining the full-length cDNA sequences, the open reading frames of TrWSD4 were amplified from total cDNAs of the protist *T. roseum* with Phusion High-Fidelity DNA Polymerase (Thermo) using the primer pair TrWSD4ec-for/TrWSD4ec-rev containing restriction sites (*Bam*HI and *Xho*I) appropriate for cloning into pMD19-T Simple vector (Takara). The fidelity of PCR was confirmed by DNA sequencing. The resulting plasmid was digested with *Bam*HI and *Xho*I, and the ORF of TrWSD4 was subcloned into the *Bam*HI and *Xho*I sites of pET23b (Novagen), generating a C-terminal His6-tagged TrWSD4 construct.

To construct the plasmid for overproduction of TrWSD5 in *E. coli*, the coding sequence of TrWSD5 was codon optimized, synthesized by GenWiz (Shanghai, China) with the deletion of a predicted C-terminal transmembrane region (residues 472–494), and amplified by PCR using the primer pair TrWSD5ec-for/TrWSD5ec-rev containing restriction sites (*Bam*HI and *Eco*RI) appropriate for cloning into pMD19-T Simple vector. The resulting plasmid was digested with *Bam*HI and *Eco*RI and the codon-optimized ΔTmTrWSD5 (missing residues 472–494) was subcloned into the *Bam*HI and *Eco*RI sites of the *E. coli* expression vector pMBP-C (BioVector, Beijing) to generate a C-terminal His_6_-tagged ΔTmTrWSD5 construct. The vectors pET23b carrying TrWSD4 and pMBP-C carrying ΔTmTrWSD5 were transformed into *E. coli* Rosetta (DE3) for recombinant enzyme overproduction. All the primers used in this study are listed in Additional file 7: Table S2.

### Construction of plasmids for heterologous expression in yeast

Full-length TrWSD4 open reading frame was amplified by PCR using the primer pair TrWSD4sc-for/TrWSD4sc-rev containing restriction sites at the 5′ ends (*Eco*RI to the sense primer and *Spe*I to the antisense primer). The PCR product was cloned into the pMD19-T Simple vector and sequenced to confirm the fidelity of the construct. Full-length cDNA of TrWSD4 in pMD19-T Simple vector was digested with *EcoR*I and *Spe*I and cloned into the *EcoR*I and *Spe*I restriction sites of the pESC-URA expression vector (Agilent Technologies).

To construct the plasmid for heterologous expression of TrWSD5 in yeast, the coding sequence of TrWSD5 was codon optimized, synthesized by GenWiz (Shanghai, China) and amplified by PCR using the primer pair TrWSD5sc-for/TrWSD5sc-rev containing restriction sites at the 5′ ends (*Eco*RI to the sense primer and *Spe*I to the antisense primer). The PCR product was cloned into the pMD19-T Simple vector and sequenced to confirm the fidelity of the construct. The codon-optimized form of TrWSD5 cDNA in pMD19-T Simple vector was digested with *Eco*RI and *Spe*I and cloned into the *Eco*RI and *Spe*I restriction sites of the pESC-URA vector.

### Enzyme overproduction in *E. coli*

For production of the TrWSD4 and TrWSD5 recombinant enzymes in *E. coli* Rosetta (DE3), starter cultures for the constructs of TrWSD4-pET23b and TrWSD5-pMBP-C were grown overnight at 37 °C in LB media containing 100 μg/mL ampicillin. Aliquots from these cultures were used to inoculate 200 mL cultures that were grown at 37 °C until mid-log phase has been reached. The cultures were induced to overexpress the recombinant WS/DGAT enzymes by the addition of 0.8 mM isopropylthio-β-galactoside (IPTG) and growth was continued for additional 6 h. The bacterial cells were harvested by centrifugation at 3000 rpm for 5 min, and lysed by sonication on ice for 15 min. Cell debris was removed by centrifugation at 12,000 rpm at 4 °C for 15 min. The clarified lysate was used to purify soluble TrWSD4–6 × His and TrWSD5–6 × His by a HisTrap FF column (GE Healthcare) according to the manufacturer’s instructions. After that, recombinant proteins were transferred to a 10 kDa molecular weight cutoff Amicon Ultra-15 centrifugal filter unit (EMD Millipore) for concentration and buffer exchange into 50 mM sodium-phosphate buffer (pH 7.4). The purified proteins were supplemented with 5% (v/v) glycerol and stored at −20 °C.

### Heterologous expression of TrWSD4 and TrWSD5 in yeast

The TrWSD4-pESC-URA and TrWSD5-pESC-URA constructs were transformed into the yeast mutant H1246 using the PEG/lithium acetate method [43]. Yeast cells transformed with the empty pESC-URA plasmid were used as control. Yeast transformants were selected by growth on synthetic complete medium lacking uracil (SC-ura), supplemented with 2% (w/v) agar and 2% glucose. The positive colonies were transferred into liquid SC-ura media supplemented with 2% (w/v) glucose and grown at 28 °C overnight. The overnight cultures were diluted to OD_600_ = 0.4 in SC-ura medium supplemented with 2% (w/v) galactose and 1% (w/v) raffinose to induce protein expression. The cultures supplemented with 0.1% (w/v) palmitic acid together with 0.1% (w/v) hexadecanol were grown at 28 °C for 48 h and then harvested by centrifugation at 3500 rpm for 5 min. The resulting pellets were washed three times with distilled water before being used for further lipid analysis.

### Assay for WS/DGAT activity

Protein concentration of the purified TrWSD4 and TrWSD5 was determined using a bicinchoninic acid protein assay kit (TIANGEN). WS/DGAT activity assays were performed using a previously described method [30]. WS activity was indirectly measured using the coupled reaction of the Ellman’s reagent [5,5′-dithio-bis(2-nitrobenzoic acid), DTNB] and free CoA released during the reaction of esterification of fatty alcohol and fatty acyl-CoA. The reaction mixture (200 μL) consisted of 25 mM sodium phosphate buffer (pH 7.4), 1 mg/mL DTNB (dissolved in DMSO), appropriate amount of purified recombinant enzymes (about 5 μg, unless otherwise specified), and the substrates of fatty alcohol and fatty acyl-CoA. Assay reactions were preincubated at 37 °C for 5 min before the reactions were initiated by the addition of the purified recombinant enzyme or acyl-CoA. The release of free CoA was monitored for additional 60 min at 412 nm (*ε* = 14,150 M^−1^ cm^−1^) on a microplate reader SpectraMax M2 (Molecular Devices). DGAT activity was determined in the same manner as described for the WS assay except that diacylglycerol (DAG, 100 μM) was used as the substrate instead of fatty alcohol. All assays were run at 37 °C, unless otherwise stated. To determine kinetic parameters for WS-catalyzed reaction, 5 μg TrWSD4 or TrWSD5 was used for each enzyme assay with specific acyl-CoA as substrate. The concentration of hexadecanol was constant at 100 μM and the concentrations of acyl-CoAs with different chain length and saturation were varied from 15 to 90 μM (15, 30, 45, 60, 75, and 90 μM). To test the effect of different temperatures on WS activity, spectrophotometric assays were conducted in 25 mM sodium phosphate buffer containing 90 μM palmitoyl-CoA and 100 μM hexadecanol as the substrates at temperatures ranging from 7 to 57 °C (7, 17, 27, 37, 47, and 57 °C). To test the effect of salt concentration on WS activity, WS assays were carried out at 37 °C in the same reaction mixture containing the substrates of 90 μM decanoyl-CoA and 100 μM hexadecanol together with different concentrations of NaCl (400, 800, 1200, and 1600 mM). WS and DGAT activities of TrWSD4 and TrWSD5 are from a representative experiment performed in triplicate.

### Determination of kinetic parameters of WS-catalyzed reaction

To obtain the kinetic constants for TrWSD4- or TrWSD5-catalyzed esterification between acyl-CoA and fatty alcohol, the standard assay was performed at 37 °C in 200 μL microplates. Initial rates (*V*_0_) were determined as the maximal slope of A_412_ against time curves using SoftMax Pro 5.3 software (Molecular Devices). The Michaelis–Menten parameters were determined by non-linear regression analysis or linear regression analysis for which the parameters were calculated according to the equation. Upon varying [S] to create saturation curves, values of *V*_0_ were fitted to the Michaelis–Menten equation *V*_0_ = *V*_max_[S]/([S] + *K*_m_) (where *V*_max_ is the maximum velocity and *K*_m_ is the Michaelis–Menten constant) using GraphPad Prism 7.0 to yield *V*_max_ and *K*_m_. For certain substrates of acyl-CoAs on which substrate inhibition was observed, the Michaelis–Menten equation was modified to *V*_0_ = *V*_max_[S]/{*K*_m_ + [S](1 + [S]/*K*_i_)}, where *K*_i_ is the inhibition constant. Values of *K*_cat_ were calculated as *V*_max_/[E].

### Measurement of acyl-CoA and fatty alcohol specificity

Acyl-CoA and fatty alcohol specificity of TrWSD4 and TrWSD5 was determined by the same spectrophotometric assays as described for WS/DGAT activity assay. Acyl-CoA specificity was measured using hexadecanol as acyl acceptor, whereas alcohol specificity was determined with palmitoyl-CoA as acyl donor. In assays for acyl-CoA specificity of TrWSD4 and TrWSD5, different molecular species of acyl-CoAs were added to the above reaction mixture. Acyl-CoA substrates used in this study include: octanoyl-Coenzyme A, decanoyl-Coenzyme A and palmitoyl-Coenzyme A which were purchased from Sigma-Aldrich, and lauroyl Coenzyme A, myristoyl Coenzyme A, stearoyl Coenzyme A, (6Z,9Z,12Z-octadecatrienoyl) Coenzyme A, (5Z,8Z,11Z,14Z-arachidonoyl) Coenzyme A, and (5Z,8Z,11Z,14Z,17Z-eicosapentaenoyl) Coenzyme A which were obtained from Avanti Polar Lipids (Alabama, USA).

### Lipid analysis by TLC and GC–MS

Yeast cells (50 mL) were harvested after 48 h of induction and washed twice with distilled water. Neutral lipids were extracted using the method described by Blight and Dyer [44]. Separation of TAG, WE, and polar lipids was performed by running silica gel 60 plates (Merck) in a developing solvent of hexane:diethylether:acetic acid (70:30:1[v/v/v]) and identified by co-migration with known standards of WE and TAG. Lipids on the TLC plate were visualized under UV light after spraying with primuline (Sigma). Spots corresponding to WEs were scraped off the TLC plates and resolved in chloroform. The wax composition was determined using gas chromatography (GC) and mass spectrometry (MS). GC (7890A; Agilent technologies) was equipped with a flame ionization detector (FID) and a HP-5 capillary column (30 m × 0.25 mm having a film thickness of 0.25 μm, Agilent technologies). The flow rate of the carrier gas (He) was constant at 1 mL min^−1^. The temperature of injector and detector was 280 °C. The column temperature was controlled at 40 °C for 2 min, raised by 20 °C min^−1^ to 300 °C, and held for 15 min at 300 °C. MS analysis was carried out with an Agilent 5795C mass spectrometer and the parameters for MS analysis were as follows: the temperature of electron ionization (EI) ion source was 230 °C, electron energy was 70 eV, and temperature of quadruples was 150 °C. The wax sample was diluted in 10 μL chloroform and 1 μL was injected into the system. Identification of wax constitutes was made on the basis of their retention times in comparison with an internal library of spectra.

## Additional files

**Additional file 1: Figure S1.** A phylogenetic tree of WS/DGATs from plant, bacteria and *T. roseum*. The tree was generated according to the Neighbor-Joining algorithm. GenBank accession numbers are shown by following the corresponding species name. The percentages of bootstrap support, calculated from 1,000 replicates, are shown on the branches.

**Additional file 2: Figure S2.** Acyl-CoA substrate specificity of WS activity of TrWSD4. Saturation curves of initial velocity (*V*_0_) for acyl-CoAs with saturated short acyl chains (**a**), saturated medium acyl chains (**b**), saturated long acyl chains (**c**), and polyunsaturated long or very long acyl chains (**d**). WS activity was determined with various fatty acyl-CoA concentrations and 100 μM hexadecanol. Reactions were performed at 37°C in mixtures including 25 mM sodium phosphate buffer (pH7.4) and 1 mg/mL DTNB.

**Additional file 3: Figure S3.** Acyl-CoA substrate specificity of WS activity of TrWSD5. Saturation curves of *V*_0_ for acyl-CoAs with saturated 8-carbon and 10-carbon acyl chains (**a**), saturated 12-carbon and 14-carbon acyl chains (**b**), saturated 16-carbon and 18-carbon acyl chains (**c**), polyunsaturated 18-carbon acyl chain (**d**), and polyunsaturated 20-carbon acyl chains (**e**). WS activity was determined with various fatty acyl-CoA concentrations and 100 μM hexadecanol. Reactions were performed at 37°C in mixtures including 25 mM sodium phosphate buffer (pH7.4) and 1 mg/mL DTNB.

**Additional file 4: Figure S4.** Saturated curves of *V*_0_ for TrWSD4 (**a**) and TrWSD5 (**b**) with 100 μM hexadecanol and different concentrations of palmitoyl-CoAs as substrates were constructed for measurements at 17°C, 27°C and 37°C. Solid lines indicate fitting of the data to the Michaelis-Menten equation.

**Additional file 5: Figure S5.** Mass spectra of wax esters produced by recombinant yeast mutant H1246 expressing the *Thraustochytrium roseum* bifunctional WS/DGAT enzyme TrWSD4 or TrWSD5. Wax esters isolated from recombinant yeast H1246 expressing TrWSD4 mainly contain hexadecyl myristate (C_14_) (molecular ion *m*/*z*=229.2, corresponding to C_30_ wax ester; **a**) and hexadecyl palmitate (C_16_) (molecular ion *m*/*z*=257.2, corresponding to C_32_ wax ester; **b**). Wax esters isolated from recombinant yeast H1246 expressing TrWSD5 are composed of hexadecyl laurate (C_12_) (molecular ion *m*/*z*=201.1, corresponding to C_28_ wax ester; **c**), hexadecyl myristate (C_14_) (molecular ion *m*/*z*=229.2, corresponding to C_30_ wax ester; **d**), and hexadecyl palmitate (C_16_) (molecular ion *m*/*z*=257.2, corresponding to C_32_ wax ester; **e**) as well as certain amounts of ethyl palmitate (molecular ion *m*/*z*=88.0, corresponding to C_16_ ethyl ester; **f**).

**Additional file 6: Table S1.** Strains and primers used in this work.

**Additional file 7: Table S2.** Comparison of characterized WS/DGATs from prokaryotes and eukaryotes.

## Abbreviations

DAG: diacylglycerol
DGAT: diacylglycerol acyltransferase
DMSO: dimethyl sulphoxide
DTNB: 5,5′-dithio-bis(2-nitrobenzoic acid)
FID: flame ionization detector
GC: gas chromatography
*k*_cat_: enzyme turnover number
*k*_cat_/*K*_m_: enzyme specificity constant
*K*_m_: Michaelis–Menten constant
MS: mass spectrometry
PCR: polymerase chain reaction
RT: reverse transcription
SDS-PAGE: sodium dodecyl sulfate–polyacrylamide gel electrophoresis
TAG: triacylglycerol
TLC: thin layer chromatography
*V*_0_: reaction velocity rate
WE: wax ester
WS: wax synthase
YPD: yeast extract peptone
