# Supplementary figures and images for: Two bifunctional enzymes from the marine protist Thraustochytrium roseum: biochemical characterization of wax ester synthase/acyl-CoA:diacylglycerol acyltransferase activity catalyzing wax ester and triacylglycerol synthesis
Source: Biotechnol Biofuels. 2017 Jul 15;10:185. doi: 10.1186/s13068-017-0869-y (PMC5513132; doi:10.1186/s13068-017-0869-y)

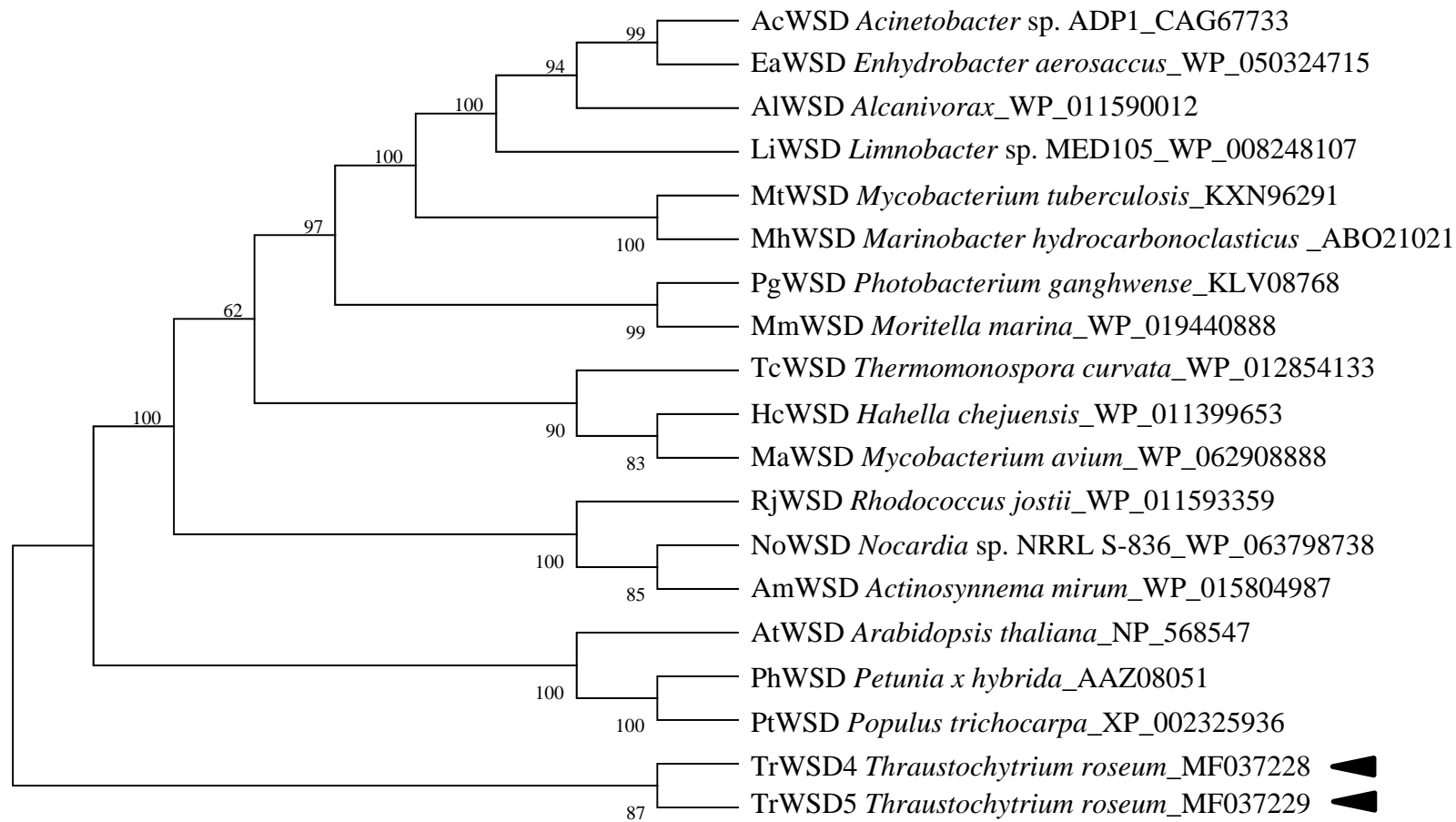

Supplement: Supplementary file 1 — Additional file 1: Figure S1. A phylogenetic tree of WS/DGATs from plant, bacteria and T. roseum. The tree was generated according to the Neighbor-Joining algorithm. GenBank accession numbers are shown by following the corresponding species name. The percentages of bootstrap support, calculated from 1,000 replicates, are shown on the branches. [file 13068_2017_869_MOESM1_ESM.pdf]

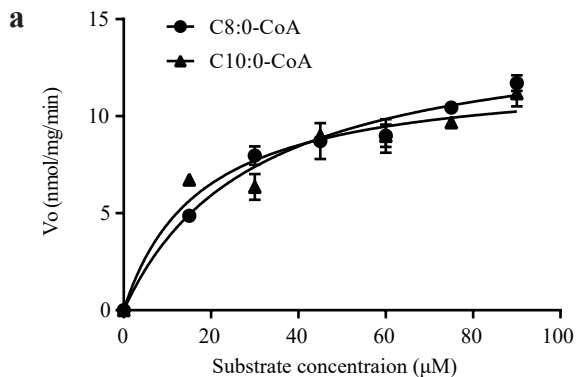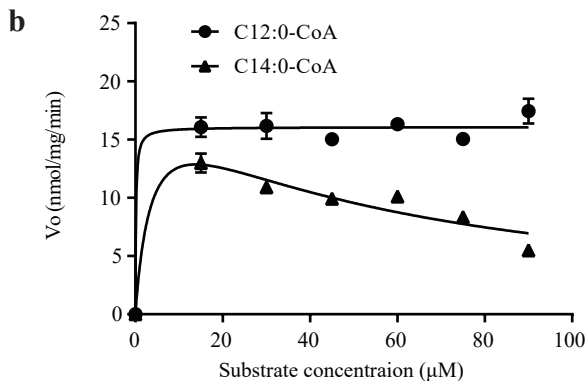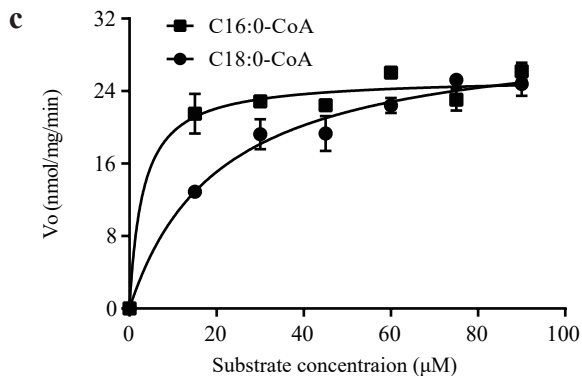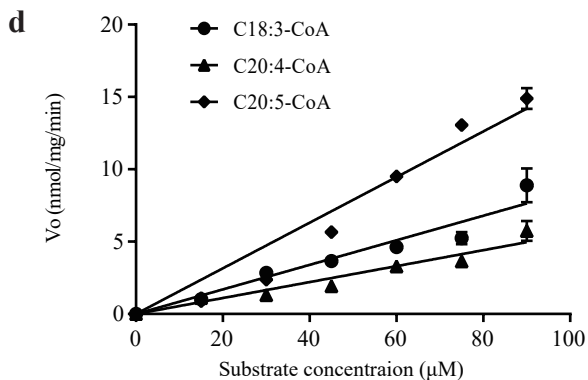

Supplement: Supplementary file 2 — Additional file 2: Figure S2. Acyl-CoA substrate specificity of WS activity of TrWSD4. Saturation curves of initial velocity (V 0) for acyl-CoAs with saturated short acyl chains (a), saturated medium acyl chains (b), saturated long acyl chains (c), and polyunsaturated long or very long acyl chains (d). WS activity was determined with various fatty acyl-CoA concentrations and 100 μM hexadecanol. Reactions were performed at 37°C in mixtures including 25 mM sodium phosphate buffer (pH7.4) and 1 mg/mL DTNB. [file 13068_2017_869_MOESM2_ESM.pdf]

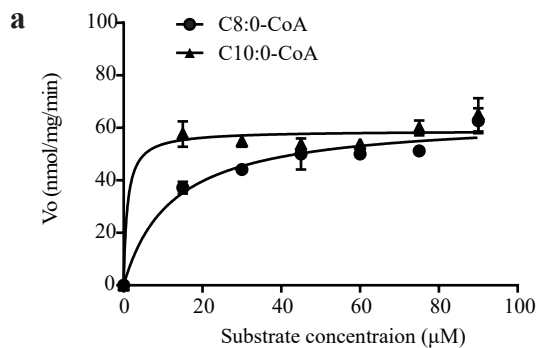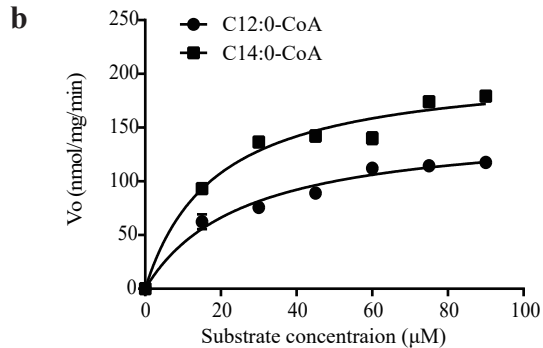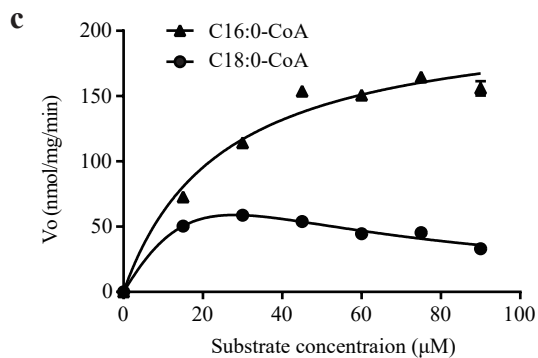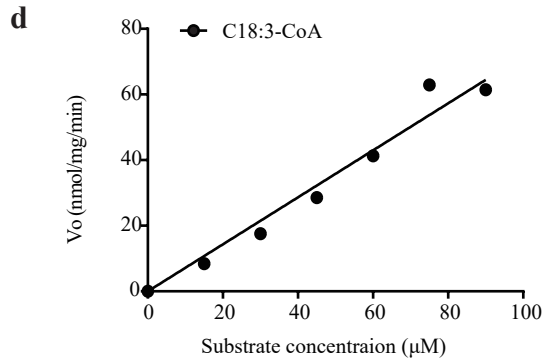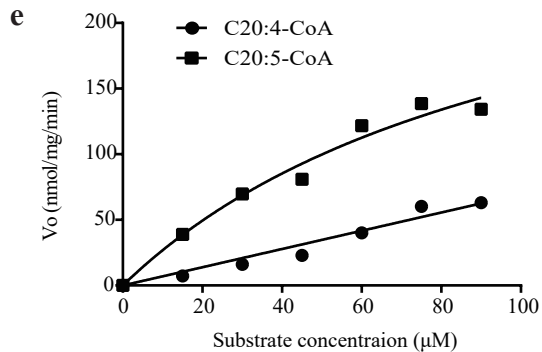

Supplement: Supplementary file 3 — Additional file 3: Figure S3. Acyl-CoA substrate specificity of WS activity of TrWSD5. Saturation curves of V 0 for acyl-CoAs with saturated 8-carbon and 10-carbon acyl chains (a), saturated 12-carbon and 14-carbon acyl chains (b), saturated 16-carbon and 18-carbon acyl chains (c), polyunsaturated 18-carbon acyl chain (d), and polyunsaturated 20-carbon acyl chains (e). WS activity was determined with various fatty acyl-CoA concentrations and 100 μM hexadecanol. Reactions were performed at 37°C in mixtures including 25 mM sodium phosphate buffer (pH7.4) and 1 mg/mL DTNB. [file 13068_2017_869_MOESM3_ESM.pdf]

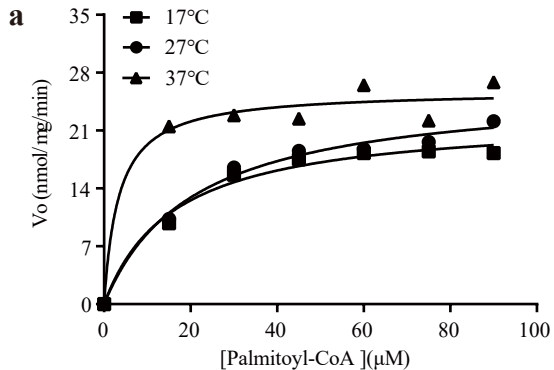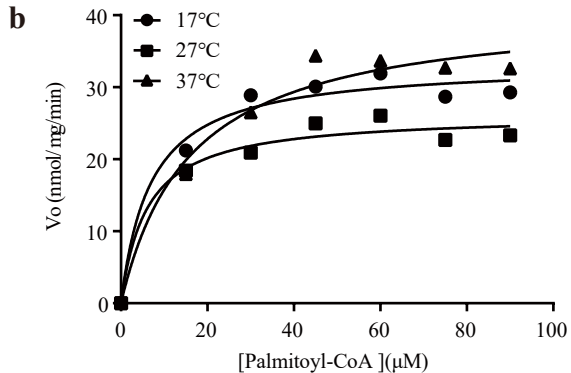

Supplement: Supplementary file 4 — Additional file 4: Figure S4. Saturated curves of V 0 for TrWSD4 (a) and TrWSD5 (b) with 100 μM hexadecanol and different concentrations of palmitoyl-CoAs as substrates were constructed for measurements at 17°C, 27°C and 37°C. Solid lines indicate fitting of the data to the Michaelis-Menten equation. [file 13068_2017_869_MOESM4_ESM.pdf]

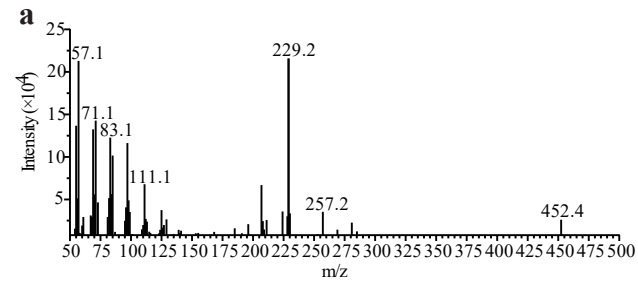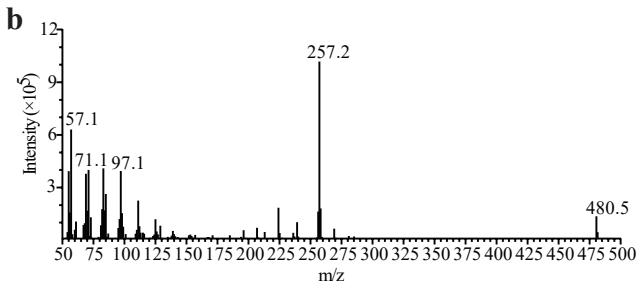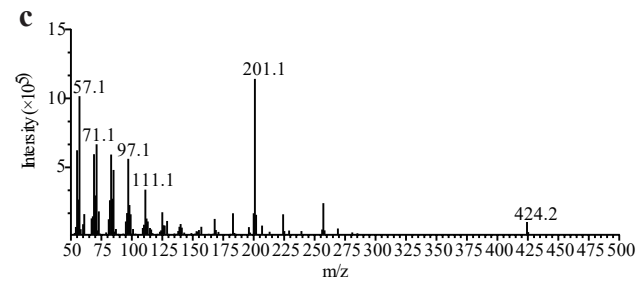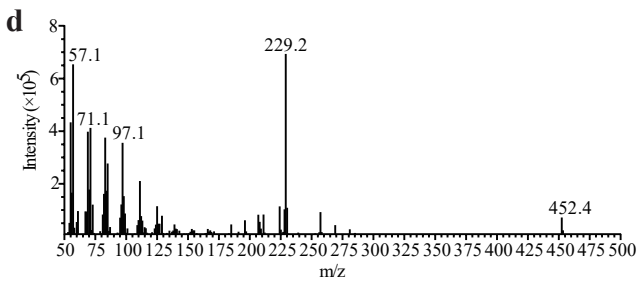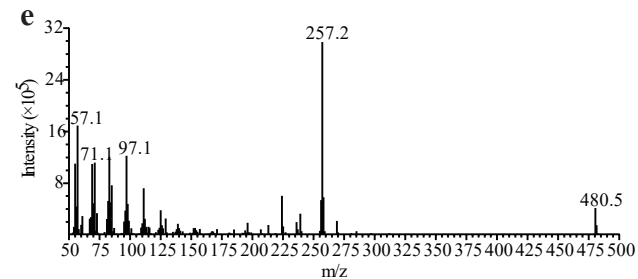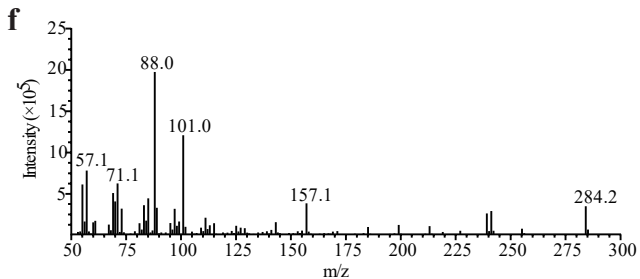

Supplement: Supplementary file 5 — Additional file 5: Figure S5. Mass spectra of wax esters produced by recombinant yeast mutant H1246 expressing the Thraustochytrium roseum bifunctional WS/DGAT enzyme TrWSD4 or TrWSD5. Wax esters isolated from recombinant yeast H1246 expressing TrWSD4 mainly contain hexadecyl myristate (C14) (molecular ion m/z=229.2, corresponding to C30 wax ester; a) and hexadecyl palmitate (C16) (molecular ion m/z=257.2, corresponding to C32 wax ester; b). Wax esters isolated from recombinant yeast H1246 expressing TrWSD5 are composed of hexadecyl laurate (C12) (molecular ion m/z=201.1, corresponding to C28 wax ester; c), hexadecyl myristate (C14) (molecular ion m/z=229.2, corresponding to C30 wax ester; d), and hexadecyl palmitate (C16) (molecular ion m/z=257.2, corresponding to C32 wax ester; e) as well as certain amounts of ethyl palmitate (molecular ion m/z=88.0, corresponding to C16 ethyl ester; f). [file 13068_2017_869_MOESM5_ESM.pdf]
