# Supplementary material for: Two bifunctional enzymes from the marine protist Thraustochytrium roseum: biochemical characterization of wax ester synthase/acyl-CoA:diacylglycerol acyltransferase activity catalyzing wax ester and triacylglycerol synthesis
Source: Biotechnol Biofuels. 2017 Jul 15;10:185. doi: 10.1186/s13068-017-0869-y (PMC5513132; doi:10.1186/s13068-017-0869-y)
Supplement: Supplementary file 6 — Additional file 6: Table S1. Strains and primers used in this work. [file 13068_2017_869_MOESM6_ESM.doc]

**Table S1** **Microbial strains and primers used in this study.**

| **Strains/primers** | **Use, relevant characteristic (s), and/or sequence (source)** |
| --- | --- |
| Strains |  |
| *E. coli* DH5α | *E. coli* host for DNA manipulations, TransGen (Beijing, China) |
| *E. coli* Rosetta (DE3) | *E. coli* host for overproduction of recombinant protein |
| *S. cerevisiae* mutant H1246 | *MATα ADE2-1 can1-100 ura3-1 are1-Δ::HIS3 are2-Δ::LEU2 dga1-Δ::KanMX4 lro1-Δ::TRP1*, kindly provided by Prof. Antoni Banas, University of Gdansk and Medical University of Gdansk, Gdansk, Poland |
| H1246:TrWSD4 | H1246 expressing TrWSD4 |
| H1246:TrWSD5 | H1246 expressing TrWSD5 |
| H1246:pESC-URA | H1246 harboring the empty pESC-URA plasmid |
| Primers |  |
| TrWSD4ec-for | 5' GGATCCGATGGCGGAGAACAACAACC 3' (*BamH*I) |
| TrWSD4ec-rev | 5' CTCGAGAAGGCAGTCCAGGCAGG 3' (*Xho*I) |
| TrWSD5ec-for | 5' CGCGGATCCATGGGCAGCGGTAAAAGCC 3' (*BamH*I) |
| TrWSD5ec-rev | 5' CCGGAATTCCAGTAACGGCTTATCTTGCTTC 3' (*EcoR*I) |
| TrWSD4sc-for | 5' GAATTCATGGCGGAGAACAACAACC 3' (*EcoR*I) |
| TrWSD4sc-rev | 5' ACTAGTGCAAGGCAGTCCAGGCAGGA 3' (*Spe*I) |
| TrWSD5sc-for | 5' GAATTCATGGGTTCTGGTAAGTCTCCAAAG 3' (*EcoR*I) |
| TrWSD5sc-rev | 5' ACTAGTGCCAACAACAAAACAGACAACAAGAT 3' (*Spe*I) |
