# Supplementary material for: Two bifunctional enzymes from the marine protist Thraustochytrium roseum: biochemical characterization of wax ester synthase/acyl-CoA:diacylglycerol acyltransferase activity catalyzing wax ester and triacylglycerol synthesis
Source: Biotechnol Biofuels. 2017 Jul 15;10:185. doi: 10.1186/s13068-017-0869-y (PMC5513132; doi:10.1186/s13068-017-0869-y)
Supplement: Supplementary file 7 — Additional file 7: Table S2. Comparison of characterized WS/DGATs from prokaryotes and eukaryotes. [file 13068_2017_869_MOESM7_ESM.docx]

**Table S2Overview of biochemical properties of characterized WS/DGATs from prokaryotes and eukaryotes.**

| Source organism& protein name | NCBI Accession Number | Number of predicted TMD(s)^a^ | Substrate spectrum of acyl-CoAs | Substrate spectrum of alcohols/acylglycerides | Other biochemical properties | Reference(s) |
| --- | --- | --- | --- | --- | --- | --- |
| Prokaryotes |  |  |  |  |  |  |
| *Acinetobacter sp.* Strain ADP1 AtfA | YP_045555 | 3 | Saturated and unsaturated C_2_−C_20_-CoA | C_2−_C_30_ linear alcohols, branched alcohols, cyclic or aromatic alcohols; mono- and diacylglycerides | The highest WS and DGAT activities could be measured at 45^o^C; The highest WS activity was measured with C16-CoA and linear C14 to C18 fatty alcohols; Exhibiting 10-fold-higher WS activitythan DGAT activity and aromatic substrates activity | [5][36][37]  [39] |
| *Marinobacteraquaeolei* Ma1 | YP_957462 | 0 | C_8_−C_16_-CoAs | C_8_−C_18_ linear alcohols, branched and aromatic alcohols | Exhibiting the highest in vivo WS activity towards C_11_ and C_12_-alcohols and C_14_-CoA | [22] |
| *Marinobacteraquaeolei* Ma2 | YP_960328 | 0 | C_8_−C_16_-CoAs | C_8_−C_18_ linear alcohols, branched and aromatic alcohols | Exhibiting the highest in vivo WS activity towards C_11_ and C_12_-alcohols and C_14_-CoA | [22] |
| *Marinobacterhydrocarbonoclasticus*WS1 | EF219376 | 0 | >C_14_ long-chain acyl-CoAs | C_10_−C_16_ fatty alcohols, isoprenoid alcohols | ShowingDGATactivity | [30] |
| *Marinobacterhydrocarbonoclasticus*WS2 | EF219377 | 0 | >C_14_ long-chain acyl-CoAs | C_10_−C_16_ fatty alcohols, isoprenoid alcohols | Showing no DGAT activity; Having a higher preference for longer-chain alcohols than WS1; Producing biodiesel in recombinant yeast; | [30] |
| *Alcanivoraxborkumensis*AtfA1 | YP_694462 | 0 | --^b^ | Short, medium, and long-chain-linear alcohols, cyclic or phenolic alcohols | Exhibitinghigh DGAT activity;Showing the highest specificity for medium-chain alcohols and a clear preference for 1-MAG^c^ than for other MAGs | [40] |
| *Rhodococcusjostii* Rh1 | WP_011594556 | 0 | C_8_−C_16_-CoAs | C_8_−C_18_ linear alcohols, branched and aromatic alcohols | Showing the highest in vivo WS activity towards C_11_ and C_12_-alcohols and C_14_-CoA | [22][40] |
| *Rhodococcusopacus* Atf2 | GQ923887 | 0 | -- | C_2_−C_18_linear alcohols | Exhibiting a low WS and DGAT activity | [38] [39] |
| *Psychrobactercryohalolentis* Ps1 | YP_579515 | 0 | C_8_−C_16_-CoAs | C_8_−C_18_ linear alcohols, branched and aromatic alcohols | Showing the highest in vivo WS activity towards C_11_, C_12_ and C_14_-alcohols and C_14_-CoA | [22] |
| Eukaryotes |  |  |  |  |  |  |
| *Petunia petals* PhWS1 | DQ093641 | 1 | C_14_−C_22_-CoAs | C_4_−C_14_ linear alcohols, benzyl alcohol | Showing WS but lacking DGAT activity inyeast;Displaying a preference for medium-chain alcohols; The highest activity was obtained at pH 7.1. | [35] |
| *Arabidopsis thaliana* WSD1 | NP_568547 | 1 | -- | C_18_, C_24_ and C_28_ alcohols | Lacking DGAT activity in recombinant yeast; Showing a very low DGAT activity in crude extracts of recombinant *E. coli* | [34] |
| *Simmondsiachinensis*ScWS | AF149919 | 7−9 | C_10_−C_22_ saturated and monounsaturated acyl-CoAs | C_10_−C_24_ saturated and unsaturated linear alcohols | Exhibiting the highest in vitro activity towards 18:0-CoA followed by 20:1-CoA and 22:1-CoA;  Showing the highest WS activity at 35^o^C and 40^o^C | [25] |
| *Mus musculus*MmWS | AY611032 | 1 | -- | C_2_−C_18_linear alcohols | Showing the highest WS activity towards long- and medium-chain alcohols ( C_10_−C_18_); Showing little or no DGAT activity in HEK 293 cells | [38] |
| *Psychrobacterarcticus*PaWS | YP_263530 | 0 | -- | C_2_−C_18_linear alcohols | Showing a relatively high WS activity towards C_12_−C_18_alcohols when compared to alcohols with carbon chain length less than 10 carbons | [38] |
| *Tetrahymenathermophila* TtWS4 | XP_001017939 | 1 | C_12_−C_18_ saturated acyl-CoAs | C_8_−C_18_linear alcohols, phytol, farnesol | Havingboth WS and DGAT activities in recombinant yeast; Showing the highest WS activity towards 18:0-CoA and 10:0-OH | [41] |
| *Thraustochytriumroseum*TrWSD4 | MF037228 | 0 | C_8_−C_20_ saturated and polyunsaturated acyl-CoAs | C_10_−C_18_linear alcohols | Displaying both WS and DGAT activities in vitro but lacking DGAT activity in recombinant yeast; Showing a clear preference for 12:0-OH; Exhibiting the highest WS activity at 47^o^C | This study |
| *Thraustochytriumroseum*TrWSD5 | MF037229 | 1 | C_8_−C_20_ saturated and polyunsaturated acyl-CoAs | C_10_−C_18_linear alcohols | Displaying both WS and DGAT activities in vitro but lacking DGAT activity in recombinant yeast; Showing a clear preference for 10:0-OH; Exhibiting the highest WS activity at 37^o^C | This study |

^a^ Abbreviations: TMD, transmembrane domain.

^b^No available information.

^c^Abbreviations: MAG, monoacylglycerol.
